# High-energy focussed extracorporeal shockwave therapy reduces pain in plantar fibromatosis (Ledderhose’s disease)

**DOI:** 10.1186/1756-0500-5-542

**Published:** 2012-10-02

**Authors:** Karsten Knobloch, Peter M Vogt

**Affiliations:** 1 Plastic, Hand and Reconstructive Surgery, Burn Center, Hannover Medical School, Carl-Neuberg-Str. 1, Hannover 30625, Germany; 2 Plastic, Hand- and Reconstructive Surgery, Hannover Medical School, Hannover Germany

**Keywords:** Extracorporeal shockwave therapy (ESWT), Fibromatosis, Ledderhose, Pain

## Abstract

**Background:**

Plantar fibromatosis is a benign disease creating nodules on the medial plantar side of affected patients. While surgical removal is regarded as the therapeutic mainstay, recurrence rates and impairment of daily activities remains substantial. High-energy focussed extracorporeal shockwave therapy has been suggested to be potentially effective in plantar fibromatosis in terms of pain reduction.

**Hypothesis:**

High-energy focussed extracorporeal shockwave therapy reduces pain in plantar fibromatosis.

**Findings:**

A total number of six patients (5 males, 58±4 years) were included with plantar fibromatosis (Ledderhose’s disease) associated with pain. Three patients were operated on previously, one had concomitant Dupuytren’s contracture. High-energy focussed ESWT was applied using a Storz Duolith SD1 (2000 impulses, 3 Hz, 1.24 mJ/mm2) in two sessions with 7 days between. Pain was 6±2 at baseline, 2±1 after 14 days and 1±1 after 3 months. Softening of the nodules was noted by all patients. No adverse effects were noted.

**Conclusions:**

High-energy focussed extracorporeal shockwave energy reduces pain in painful plantar fibromatosis (Morbus Ledderhose). Further large-scale prospective trials are warranted to elucidate the value of high-energy focussed extracorporeal shockwave therapy (ESWT) in plantar fibromatosis in terms of recurrence and efficacy.

## Findings

Plantar fibromatosis is named after the German surgeon Prof. Dr. Georg Ledderhose following his report on a Dupyutren-like disease of the foot sole in 1897. As it is the case for Dupuytren’s disease of the palm, aetiology remains largely unknown to date [1]. A familiar disposition might account for a higher risk. Furthermore, patients suffering from Dupuytren’s contracture may simultaneously present with plantar fibromatois, knuckle pads on the dorsum of the fingers and penile fibromatosis (Peyronie’s disease).

Similar to Dupuytren’s disease of the hand, where in early stages nodules are evident, plantar fibromatosis yields 1-2cm nodules located typically on the medial side of the foot arch. Therapeutically, radiotherapy as well as surgery have been applied in a number of cohort studies and case series with inconsistent results. A cohort study from the Netherlands reported on 27 patients with plantar fibromatosis who underwent 40 operations on 33 feet [2]. The overall recurrence rate was 60%. Combining surgery and radiotherapy was studied in another report. However, as stated by the authors, radiotherapy was associated with a significantly impaired functional status in 50% of the patients [3]. A German cohort report with 25 patients found 44% less nodules 38months following 30Gy radiotherapy [4]. Besides, pain was reduced in 60% of the patients. Adverse effects reported were dry skin and slight erythema following radiotherapy. To date, the role of alternative therapeutic options in Ledderhose’s disease is undetermined.

Penile fibromatosis referred to as Peyronie’s disease affects middle-aged men between 40 and 60 years with penile pain, curvature during erection and potentially erectile dysfunction. Extracorporeal shockwave therapy (ESWT), which was introduced from urologists in order to destroy kidney stones, has been tested in penile fibromatosis (Evidence level Ib) [5]. Another study reported that about half of all ESWT treated patients had a significant reduction in penile angulation [6]. Given these observations in penile fibromatosis, we started to perform a randomized-controlled trial to evaluate high-energy focussed ESWT in palmar fibromatosis, Dupuytren’s contracture (DupuyShock) [7].

Given the similiarities of penile and plantar fibromatosis in a histological and clinical perspective, we sought to evaluate the effects of high-energy focussed ESWT on plantar fibromatosis in a pilot series, which is presented herein.

### Research hypothesis

Given the aforementioned effect in penile fibromatosis, we hypothesize that high-energy focussed extracorporeal shock wave therapy is able to reduce pain in plantar fibromatosis (Ledderhose’s disease).

## Methods

**Figure 1:**
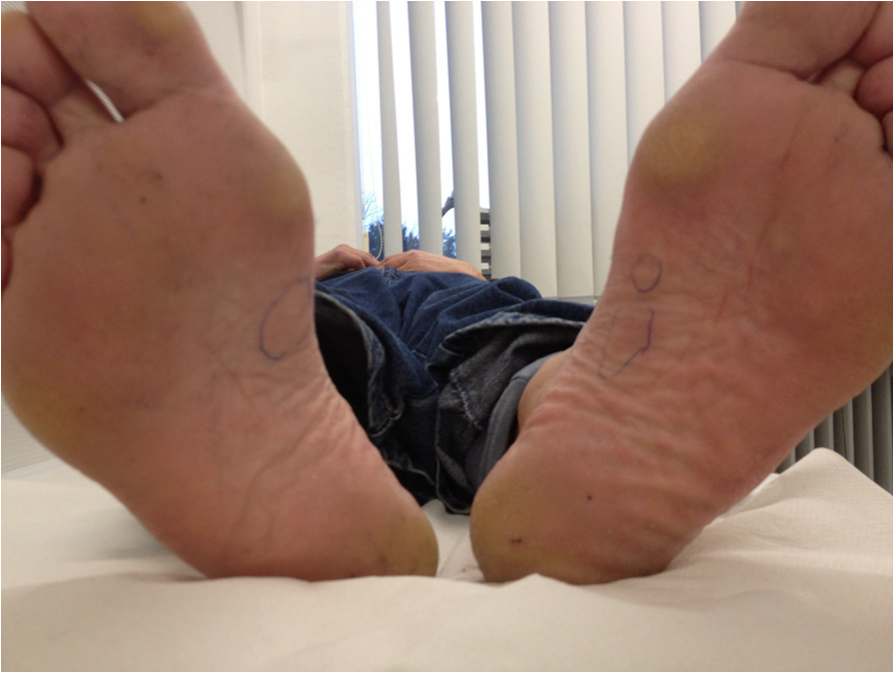
**Plantar fibromatosis as Ledderhose’s disease in a bilateral setting in a male patient with concomitant severe bilateral Dupuytren’s *contracture.***

**Figure 2:**
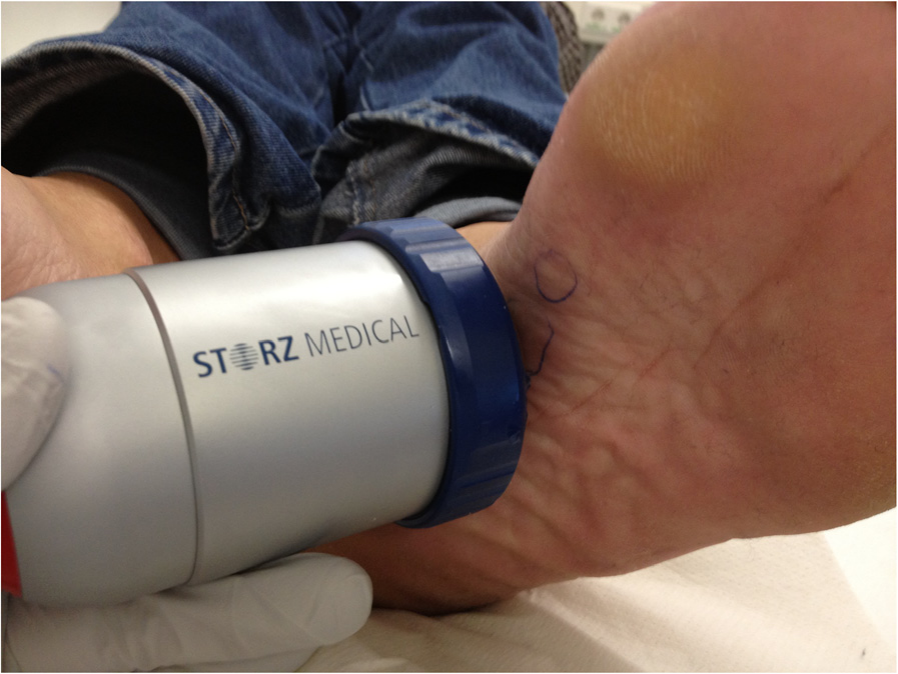
High-energy focussed extracorporeal shockwave therapy (ESWT, Storz Duolith SD1) in plantar fibromatosis (Ledderhose’s disease).

High-energy focussed ESWT was applied without anesthesia using a Storz Duolith SD1 device in two therepeutic sessions with a week interval in between with the following settings (Figures 1 and 2):

● Number of shockwaves: 2000 impulses

● Frequency: 3 Hertz

● Energy flux density 1.24 mJ/mm^2^

Patients were allowed to walk or sport following ESWT therapy. All patients documented pain in the morning on a visual analogue scale (VAS 0–10) on a daily basis.

## Results

**Figure 3:**
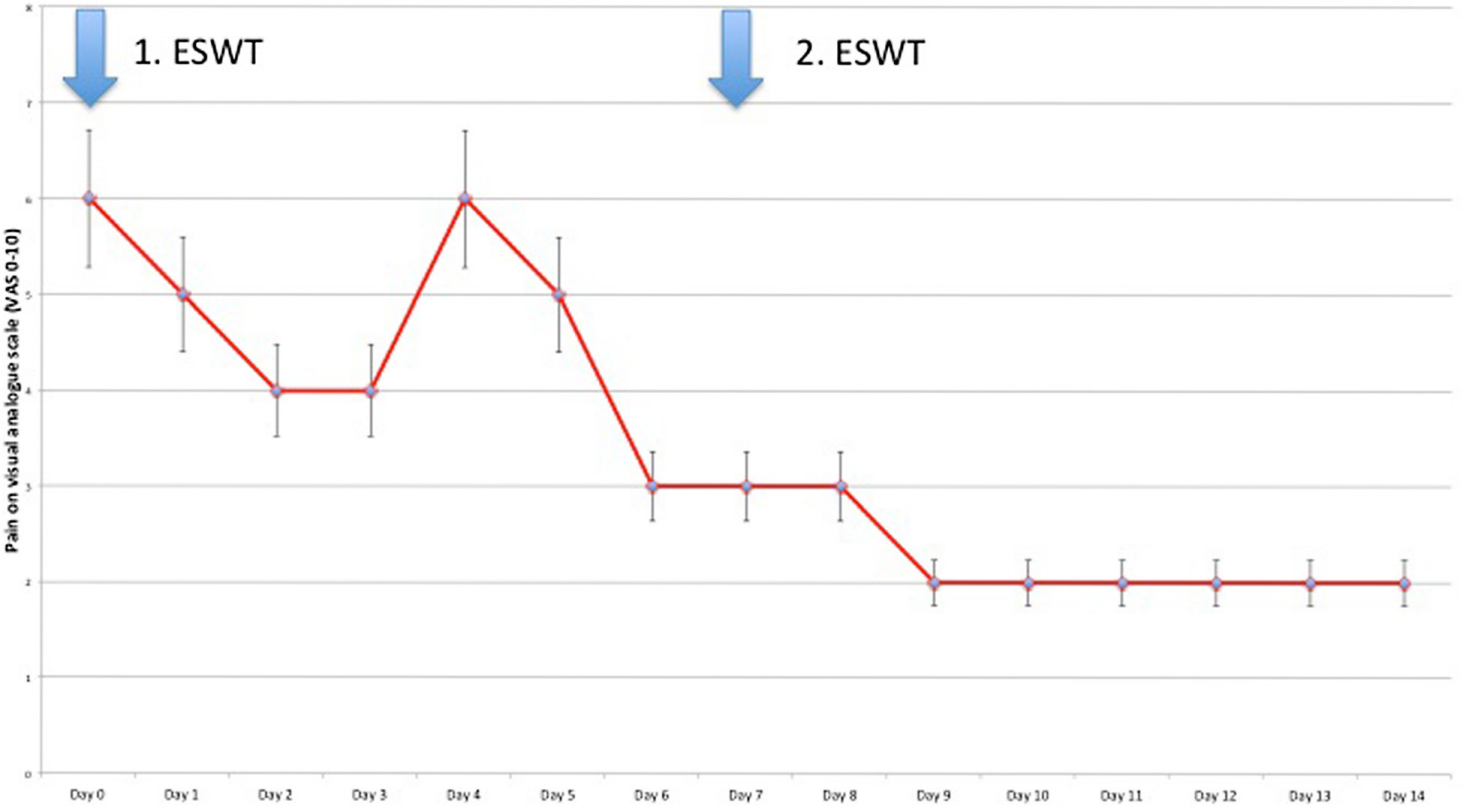
Pain daily log during two high-energy focussed ESWT sessions at day 0 and day 7.

A total number of six patients (5 males, 58±4 years) were included with plantar fibromatosis associated with pain. Three patients were operated on before. High-energy focussed ESWT was applied using a Storz Duolith SD1 (2000 impulses, 3 Hz, 1,25 mJ/mm2) for 1–3 sessions. Pain was 6±2 at baseline for at mean 4±2 months and 2±1 after 14 days (p<0,05, Figure 3). At three months follow-up, pain was 1±1. Softening of the nodules was noted by all patients. No adverse effects were noted. We found no meaningful difference in the outcome of Ledderhose patients with or without previous plantar surgery.

## Conclusion

High-energy focussed extracorporeal shockwave energy reduces pain in painful plantar fibromatosis. Further large-scale prospective trials are warranted to elucidate the value of high-energy focussed extracorporeal shockwave therapy in plantar fibromatosis in terms of recurrence and efficacy.

## Consent

Written informed consent was obtained from the patient for publication of this report and any accompanying images.

## Ethical approval

This pilot case series was in compliance with the Helsinki Declaration. This pilot case series was done with an approved CE-certified medical device and the local ethical committee gave approval as case series.

## Competing interests

KK has received speaker honoraries by Pfizer and Storz. PMV has nothing to disclose.

## Authors' contribution

KK has treated the patients, documented the cases and published it. PMV has co-composed the manuscript and discussed the case. All authors read and approved the final manuscript

## References

[B1] Zgonis T Jolly GP Plyzois V Kanuck DM Stamatis ED Plantar fibromatosis Clin Podiatr Med Surg 2005 22 1 11 18 10.1016/j.cpm.2004.08.002 15555840

[B2] Van der Veer WM Hamburg SM de Gast A Niessen FB Recurrence of plantar fibromatosis after plantar fasciectomy: single-center long-term results Plast Reconstr Surg 2008 122 2 486 491 10.1097/PRS.0b013e31817d61ab 18626366

[B3] De Bree E Zoetmulder FA Keus RB Peterse HL van Coevorden F Incidence and treatment of recurrent plantar fibromatosis by surgery and postoperative radiotherapy Am J Surg 2004 187 1 33 38 10.1016/j.amjsurg.2002.11.002 14706583

[B4] Seegenschmiedt MH Attassi M Radiation therapy for Morbus Ledderhose – indication and clinical results Strahlenther Onkol 2003 179 12 847 853 10.1007/s00066-003-0994-3 14652674

[B5] Palmieri A Imbimbo C Longo N Fusco P Verze F Mangiopia F A first prospective, randomized, double-blind, placebo-controlled clinical trial evaluating extracorporal shock wave therapy for the treatment of Peyronie’s disease Eur Urol 2009 56 2 363 369 10.1016/j.eururo.2009.05.012 19473751

[B6] Srirangam SJ Manikandan R Hussain J Collins GN O’Reilly PH Long-term results of extracorporeal shockwave therapy for Peyronie’s disease J Endourol 2006 20 11 880 884 10.1089/end.2006.20.880 17144855

[B7] Knobloch K Kuehn M Vogt PM Focussed extracorporeal shockwave therapy in Dupuytren’s disease-a hypothesis Med Hypotheses 2011 76 5 635 637 10.1016/j.mehy.2011.01.018 21277691

